# Solid-Contact Ion-Selective Electrodes: Response Mechanisms, Transducer Materials and Wearable Sensors

**DOI:** 10.3390/membranes10060128

**Published:** 2020-06-23

**Authors:** Yan Lyu, Shiyu Gan, Yu Bao, Lijie Zhong, Jianan Xu, Wei Wang, Zhenbang Liu, Yingming Ma, Guifu Yang, Li Niu

**Affiliations:** 1 School of Civil Engineering, c/o Center for Advanced Analytical Science, School of Chemistry and Chemical Engineering, Guangzhou University, Guangzhou 510006, China; yanlyu@e.gzhu.edu.cn (Y.L.); baoyu@gzhu.edu.cn (Y.B.); ccljzhong@gzhu.edu.cn (L.Z.); wangw@gzhu.edu.cn (W.W.); cczbliu@gzhu.edu.cn (Z.L.); ccymma@gzhu.edu.cn (Y.M.); 2 State Key Laboratory of Electroanalytical Chemistry, c/o Engineering Laboratory for Modern Analytical Techniques, Changchun Institute of Applied Chemistry, Chinese Academy of Sciences, Changchun 130022, China; jnxu@ciac.ac.cn; 3 University of Chinese Academy of Sciences, Beijing 100039, China; 4 School of Information Science and Technology, Northeast Normal University, Changchun 130117, China; young@nenu.edu.cn; 5 MOE Key Laboratory for Water Quality and Conservation of the Pearl River Delta, Guangzhou University, Guangzhou 510006, China

**Keywords:** ion selective electrodes, wearable sensors, solid-contact materials, response mechanism

## Abstract

Wearable sensors based on solid-contact ion-selective electrodes (SC-ISEs) are currently attracting intensive attention in monitoring human health conditions through real-time and non-invasive analysis of ions in biological fluids. SC-ISEs have gone through a revolution with improvements in potential stability and reproducibility. The introduction of new transducing materials, the understanding of theoretical potentiometric responses, and wearable applications greatly facilitate SC-ISEs. We review recent advances in SC-ISEs including the response mechanism (redox capacitance and electric-double-layer capacitance mechanisms) and crucial solid transducer materials (conducting polymers, carbon and other nanomaterials) and applications in wearable sensors. At the end of the review we illustrate the existing challenges and prospects for future SC-ISEs. We expect this review to provide readers with a general picture of SC-ISEs and appeal to further establishing protocols for evaluating SC-ISEs and accelerating commercial wearable sensors for clinical diagnosis and family practice.

## 1. Introduction

With the rapid growth of personal healthcare and fitness systems, wearable devices that can provide real-time and continuous monitoring of an individual’s physiological state have attracted great attention in recent years. Conventional applications in medical biomarker detection generally related to separated collection and analysis of blood samples is invasive and costly and needs complicated operations, while failing to provide users with real-time health diagnostics and monitoring. Wearable sensors hold great promise for continuously monitoring an individual’s physiological bio-chemical signals [1,2,3,4,5].

A variety of flexible sensors have recently been developed for non-invasively assessing personal physiological states by detecting analytes of interest (like those in sweat) [6,7,8,9]. Potentiometric sensors, particularly for the ion sensing, are one of the attractive types for practical application due to their high portability, good sensitivity, lower energy consumption and high efficiency. Potentiometry incorporates a working electrode (WE) and a reference electrode (RE) and measures their relative potential under zero current. Ion-selective electrodes (ISEs) are the typical potentiometric sensor for selective ion recognition.

**Figure 1:**
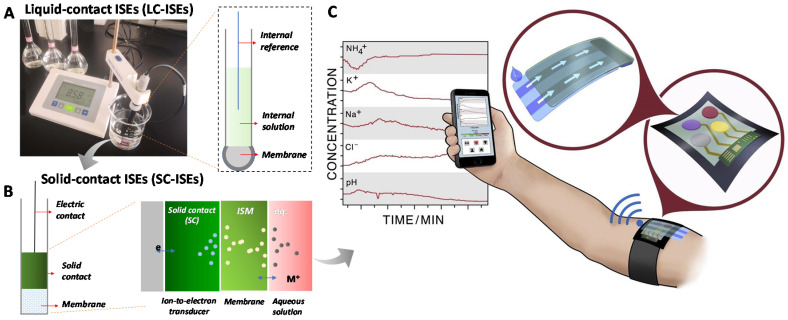
An overview from liquid-contact ion-selective electrodes (LC-ISEs) to solid-contact ISEs (SC-ISEs) for wearable sensors. (**A**) Classic LC-ISEs (e.g., pH meter) by a liquid-contact between internal solution and ion-selective membrane (ISM). (**B**) The structure of SC-ISEs by a solid-contact between solid ion-to-electron transducer layer and ISM. (**C**) An example of the SC-ISEs for wearable sensor applications [9].

Classic liquid-contact ISEs (LC-ISEs) contain an ion-selective membrane (ISM, e.g., pH glass membrane) and an internal solution (Figure 1A) to form a liquid–contact interface [10]. The theoretical fundamental of ISEs is based on the relationship between ion activity and output voltage according to the Nernst equation. The electromotive force (EMF) is the sum of all the phase boundary potentials. LC-ISEs have been commercialized and quite popular for various ion analysis in the laboratory and environmental analysis. However, the biggest challenge for the LC-ISEs is miniaturization and integration, which could not satisfy the requirements in biological relative applications, like cell or tissue-level ion analysis and wearable sensors. Cattrall and Freiser fabricated the first solid-contact ISEs (SC-ISEs) without the internal solution in 1971, which they called “coated wire electrodes” (CWEs) [11]. A quite simple electrode structure was proposed, i.e., a metal wire directly coated with Ca^2+^ ionophore-containing polymeric sensing membrane. The CWEs exhibited the exciting Nernstian response toward Ca^2+^. Freiser et al. further extended the CWEs for other ions [12]. Although the large potential drift is attributed to the unstable potential at the metal/membrane interface, the milestone CWEs opened the way for the SC-ISEs [13,14,15].

In 1992, Lewenstam and Ivaska et al. further focused on this issue and proposed an intermediate polypyrrole (PPy) [16] solid-contact layer between an ISM and conducting substrate, called the “ion-to-electron transducer layer” (Figure 1B). This transducer can transfer the ion concentration to electron signal and stabilizes the potential at the substrate/ISM interface. This electrode structure has become the-state-of-the-art standard model of SC-ISEs. Currently, numerous materials have been developed for the transducer, such as conducting polymers, multifarious carbon materials, nanomaterials, molecular redox couples etc. The potential stability has been remarkably improved and the applications for SC-ISEs are also attracting increasing attention. A representative application is for wearable sensors [9] (Figure 1C). Since the all-solid-state structure is the characteristic for the SC-ISEs, this feature makes the SC-ISEs flexible, stretchable and miniaturized.

This review concentrates on the recent advances of SC-ISEs for wearable sensors in real-time and non-invasive analysis of ions in biological fluids. Before that, we will illustrate the response mechanisms and the crucial solid-contact transducer materials. In the response mechanism section, the two mechanisms including redox capacitance and electric-double-layer (EDL) capacitance-based potential stabilization will be discussed. For the solid-contact transducer materials, classic conductive polymers and carbon materials and other nanomaterials (e.g., Au nanomaterials) will be illustrated in detail. In the wearable sensor sections, we will mainly discuss the flexible SC-ISEs for real-time monitoring ion in biological fluids, mainly sweat ion analysis. Finally, we summarize the current developments, existing challenges, and future prospects for SC-ISEs-based portable and wearable sensors.

## 2. Response Mechanisms

**Figure 2:**
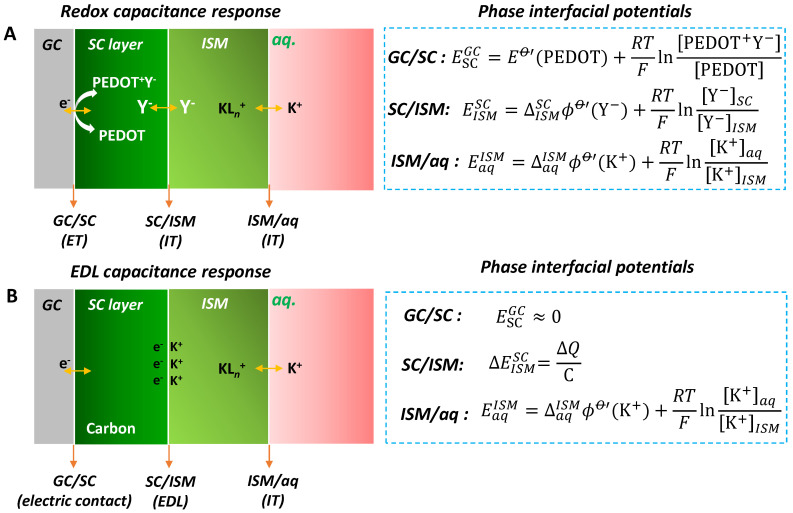
Response mechanisms for the SC-ISEs. (**A**) Redox capacitance-based SC-ISEs with poly(3,4-ethylenedioxythiophene) (PEDOT) as an example for redox SC transducer. (**B**) Electric-double-layer (EDL) capacitance-based SC-ISEs with carbon as an example for EDL SC transducer. Both SC-ISEs contain three interfaces, GC/SC, SC/ISM and ISM/aq. GC: glass carbon electrode substrate; SC: solid contact; aq: aqueous solution; ET: electron transfer; IT: ion transfer. The corresponding phase interfacial potentials are presented on the right (detailed illustration shown in the main text and Appendix A).

Like the conventional LC-ISEs, the response mechanism for the SC-ISEs is the sum of the phase interfacial potentials. As shown in Figure 2, there are two typical response mechanisms [14] for the ion-to-electron single transformation including the redox capacitance mechanism (Figure 2A) and the EDL capacitance mechanism (Figure 2B). These two mechanisms have been verified experimentally [17,18]. Herein we briefly illustrate the mechanisms through the phase interfacial potential. The response mechanism generally depends on the SC materials.

***Redox capacitance mechanism***. For the redox capacitance-based SC-ISEs, the SC materials disclose highly reversible redox behavior and can effectively attach on the surface of the substrate electrode, for example, the typical poly(3,4-ethylenedioxythiophene) (PEDOT) redox conductive polymer. These materials exhibit both electric and ionic conductivities. They convert the target ion concentration to an electron signal through the oxidation or reduction reaction. Taking the Y^−^ anion-doped PEDOT and K^+^ response as an example (Figure 2A), the ion-to-electron response process can be described as follows:

(1) $${\mathrm{PEDOT}}^{+}Y^{-}\left(\mathrm{SC}\right)+K^{+}\left(aq\right)+e^{-}\left(\mathrm{GC}\right)⇌\mathrm{PEDOT}\left(\mathrm{SC}\right)+Y^{-}\left(\mathrm{ISM}\right)+K^{+}\left(\mathrm{ISM}\right)$$

where PEDOT^+^Y^−^ (SC) and PEDOT (SC) represent the oxidation and reduced states of PEDOT in the SC phase, respectively; K^+^ (aq) and K^+^ (ISM) are the concentrations in the measured aqueous solution and ISM phase, respectively; Y^−^ (SC) and e^−^ (glass carbon, GC) present the Y^−^ in the SC and GC electrode phases, respectively.

It should be noted that the above Equation (1) represents the overall ion-to-electron reaction. In fact, the overall reaction involves three equilibrium charge transfers at three interfaces (or phase boundary). The first is the electron transfer (ET) of PEDOT at the GC/SC interface. The GC/SC interfacial potential $E_{\mathrm{SC}}^{\mathrm{GC}}$ can be described according to the Nernst equation,

(2) ESCGC=EO′(PEDOT)+RTFln[PEDOT+Y−][PEDOT]

where EO′(PEDOT) is the conditional electrode potential of PEDOT; *R*, *T* and *F* represent the gas constant, temperature and Faradaic constant, respectively. Once the PEDOT is attached on the GC electrode, their concentrations are fixed, leading to a constant potential of $E_{\mathrm{SC}}^{\mathrm{GC}}$.

The second equilibrium charge transfer is the ion transfer (IT) of doped Y^−^ anion at the SC/ISM interface. For example, taking the tetrakis-(pentafluorophenyl)borate (TPFPhB^−^) anion-doped PEDOT as an example, the TPFPhB^−^ is the doped counter ion of PEDOT in the solid contact phase and also the electrolyte of KTPFPhB in the ISM phase. The TPFPhB^−^ anion in the two phases reaches the IT equilibrium state. The SC/ISM interfacial potential $E_{\mathrm{ISM}}^{\mathrm{SC}}$ can be described as follows (the detailed derivation, see Appendix A),

(3) EISMSC=ΔISMSCϕO′(Y−)+RTFln[Y−]SC[Y−]ISM

where ΔISMSCϕO′(Y−) is the conditional ion transfer potential of Y^−^ anion from SC to ISM phase; ${\left[Y^{-}\right]}_{\mathrm{ISM}}$ and ${\left[Y^{-}\right]}_{\mathrm{SC}}$ represent the Y^−^ concentrations in the ISM and SC phases, respectively. It should be noted that multi-ion transfer equilibrium could exist at the SC/ISM interface. If that happens, multi-ion transfer Nernst equations like Equation (3) should be presented. The SC/ISM interfacial potential is determined by the sum of multi-ion transfer Nernst equations. Once the SC and ISM are attached, the $Y^{-}$ concentration are fixed and the $E_{\mathrm{ISM}}^{\mathrm{SC}}$ is also a constant.

The third equilibrium charge transfer is the IT of K^+^ at the ISM/aq interface. Similar to Y^−^ at SC/ISM interface, the ISM/aq interfacial potential is described as follows,

(4) EaqISM=ΔaqISMϕO′(K+)+RTFln[K+]aq[K+]ISM

where ΔaqISMϕO′(K+) is the standard ion transfer potential of K^+^ from ISM to aqueous phase; ${\left[K^{+}\right]}_{aq}$ and ${\left[K^{+}\right]}_{\mathrm{ISM}}$ represent the K^+^ concentration in the aqueous and ISM phases, respectively. It should be noted that the IT of K^+^ dominates the ISM/aq interfacial potential. There could not be multi-ion transfer equilibrium at this interface since the ionophore can selectively recognize the K^+^.

The potential for the SC-ISEs (*E*) is the sum of all the three interfacial potentials (Equations (2)–(4)), which is described as follows,

(5) $$E=E_{\mathrm{SC}}^{\mathrm{GC}}+E_{\mathrm{ISM}}^{\mathrm{SC}}+E_{aq}^{\mathrm{ISM}}=k+\frac{RT}{F}\ln{\left[K^{+}\right]}_{aq}$$

Once the SC and ISM components are fixed, except for the ${\left[K^{+}\right]}_{aq}$, the other items (Equations (2)–(4)) are the constant (*k*). Thus, the measured potential *E* shows the Nernstian response toward the target ion. We should here emphasize that the potential for redox capacitance-based SC-ISEs is thermodynamically defined according to Equation (5).

As the most popular SC transducer materials, a wide range of conducting polymers have been explored. In addition, other redox molecule and nanomaterials with effective ion-to-electron transduction have been developed, which will be discussed in Section 3.

***EDL capacitance mechanism***. For SC-ISEs with an EDL capacitance mechanism (Figure 2B), the ion-to-electron transduction is formed from the EDL between the ISM and the SC interface. It can be described as an asymmetric capacitor where one side is formed by electrons (or holes) and the other side is balanced by ions (cations or anions) from the ISM. The interfacial potential depends on the quantity of charge and EDL capacitance. Like redox capacitance-based SC-ISEs, there are also three interfaces including GC/SC, SC/ISM and ISM/aq interfaces (Figure 2B).

For the GC/SC interface, the potential is very small ($E_{\mathrm{SC}}^{\mathrm{GC}}$ ≈ 0) since most EDL capacitance-based SC materials are highly electronic conductive, for example carbon materials. For the SC/ISM interface, this interface has no charge transfer reaction so that this interfacial potential cannot be defined. It can only determine the potential variation, which is descried as follows,

(6) $$\Delta E_{\mathrm{ISM}}^{\mathrm{SC}}=\frac{\Delta Q}{C}$$

where ΔQ is the passed charge and C is the EDL capacitance. For the ISM/aq interface, the potential is the same with Equation (3). Therefore, the potential stability for the EDL capacitance type SC-ISE depends on the SC/ISM interface. If the EDL capacitance is high, the potential change would be small or even approaches zero.

A straightforward approach to increase the EDL capacitance is increasing the interfacial contact area between the ISE membrane and the SC. For example, porous carbon materials and nanomaterials have been developed for increasing the EDL capacitance. We will discuss this in Section 3.

## 3. Transducer Materials

The development of SC transducer materials is the core subject of SC-ISEs. Conceptually, SC transducer works as an ion-to-electron signal transformation to provide operationally stable and reproducible potentials. For the wearable sensors, SC transducer materials require more properties, such as portability, excellent flexibility, and maintenance-free. The reversible and equilibrium stability on ion-to-electron transduction in the solid state requires high exchange currents compared with the current passed during the test. Numerous efforts have been devoted to exploring better SC materials with high conductivity, hydrophobicity, large capacitance, and high stability. Next, we will discuss the state-of-the-art SC transducer materials including conducting polymers (CPs), carbon-based materials and other typical nanomaterials.

### 3.1. Conducting Polymers

CPs are the most popular SC transducer materials for SC-ISEs. CPs were first introduced as efficient ion-to-electron transducers by Lewenstam and Ivaska groups in the early-1990s [16]. They proposed PPy as the SC. Nernstian response toward Na^+^ sensing was resulted, and the stability lasted over 10 days. This work has enabled the establishment of CPs-based SC-ISEs with remarkably improved stability compared with the CWEs. Over the last three decades, CPs have been intensively explored for SC materials [13,19,20,21,22,23], such as PPy, PEDOT, poly(3-octylthiophene) (POT), polyaniline (PANI) and so on. One of the featured characteristics for CPs is their mixed electronic and ionic conductivities. The ionic signal can be effectively transformed to electronic signal through the redox of CPs coupled with ion transfer (Figure 2A). In addition, the stable potentiometric signals contribute to their high redox capacitance, which overcomes the small current passed through the electrode during zero-current conditions and environmental interruption. CPs could be easily electrodeposited on the electrode substrate and controlled by the chronocoulometry. The flexibility of CPs also becomes the advantage for the wearable sensors. However, there remain several challenges for the CP-based SC-ISEs: (1) undesired electrochemical side reactions leading to environmental sensitivity, such as gas or light; (2) the accumulation of a water layer at the SC/ISM interface.

**Figure 3:**
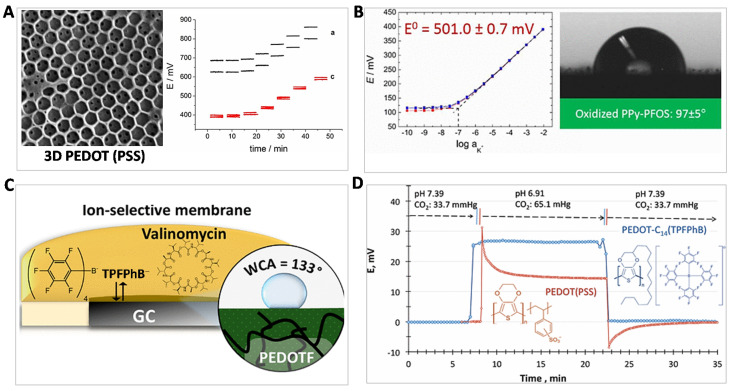
Structural design and functionalization of conducting polymers (CPs) for CP-based SC-ISEs. (**A**) Scanning electronic microscopy (SEM) image of the designed high-surface 3D poly(3,4-ethylenedioxythiophene) polystyrene sulfonate (PEDOT(PSS)) by nanosphere lithography and electrosynthesis. The right: the potential traces of 3D PEDOT(PSS) functionalized with (red line) and without (black line) a lipophilic redox 1,1′-dimethylferrocene. It is found that the functionalized 3D PEDOT(PSS) exhibits much better reproducibility. Reprinted with permission from [24], Copyright (2016) John Wiley and Sons publications. (**B**) The oxidized PPy by doping perfluorooctanesulfonate (PFOS^−^) anion (PPy-PFOS) enhanced the hydrophobicity leading to remarkably improved reproducibility. Reprinted with permission from [25], Copyright (2017) American Chemical Society. (**C**) Superhydrophobic tetrakis-(pentafluorophenyl)borate (TPFPhB^−^) anion doping PEDOT as an SC transducer to reduce the water-layer effect. It should be noted the TPFPhB^−^ ion transfer (ion exchange) between SC and ISM further enhanced the potential stability (see Equation (3)). The abbreviation of tetrakis-(pentafluorophenyl)borate on the original Figure is TFAB^−^. Herein it is replaced by TPFPhB^−^. Reprinted with permission from [26], Copyright (2019) American Chemical Society. (**D**) An ultimate approach by both C_14_-chain functionalized PEDOT and TPFPhB^−^ doping to improve the performances of SC-ISEs. Reprinted with permission from [27], Copyright (2017) American Chemical Society.

To reduce the interfacial polarization of SC/ISM, Gyurcsányi and coworkers reported 3D nanostructured poly(3,4-ethylenedioxythiophene) polystyrene sulfonate (PEDOT(PSS)) film as a SC material for Ag^+^ sensing by using 3D nanosphere lithography and electrosynthesis [24] (Figure 3A). The constructed 3D nanostructured PEDOT(PSS) film had almost 7 times larger redox capacitance than the one fabricated by direct electrosynthesis. In addition, the 3D PEDOT(PSS) was functionalized with a lipophilic redox couple (1,1′-dimethylferrocene) to further increase the redox capacitance and hydrophobicity. The EMF response revealed an improved standard deviation E^0^ of 3.9 mV. Such a 3D structure offers high redox capacitance to enhance the reproducibility of SC-ISEs.

The hydrophobicity for the classic CPs (like PEDOT and PPy) is in fact insufficient for eliminating the water layer. Tuning the counter ion doping is an efficient strategy to improve the intrinsic hydrophobicity of CPs. For example, Gyurcsányi and co-workers implemented a hydrophobic perfluorinated anion, perfluorooctanesulfonate (PFOS^−^) as a doping ion in PPy, which increased the hydrophobicity [25] (contact angle: 97° ± 5°) (Figure 3B). The PFOS^−^ ion doping resulted in an oxidized PPy that enhanced its conductivity. An improved reproducible standard potential E^0^ of 501.0 ± 0.7 mV has been achieved, which is a very small standard deviation for CP-based SC-ISEs. Very recently, Lindfors and coworkers further introduced a more hydrophobic counter ion, tetrakis-(pentafluorophenyl)borate (TPFPhB^−^) for doping PEDOT [26] (Figure 3C). The water contact angle is up to 133°. It should be noted that the TPFPhB^−^ anion exists in both SC phase and ISM phase, which determines the SC/ISM interfacial potential (see Equation (3)). By virtue of TPFPhB^−^ ion doping, the K^+^ sensor showed a low drift of 50 μV/h over 49 days.

In addition to counter ion doping, Lindner and coworkers improved the framework of PEDOT and used TPFPhB^−^ ion doping simultaneously [27] (Figure 3D). They functionalized PEDOT with a C_14_ alkane chain (PEDOT-C_14_). The superhydrophobic counter ion, TPFPhB^−^ was also doped into PEDOT-C_14_. This strategy further improved the interfacial hydrophobicity (contact angel: 136° ± 5°). This SC material has been fabricated for pH, K^+^ and Na^+^ sensors. The long-term potential stability showed the lowest drift (0.02 ± 0.03 mV/day). For the pH response, it disclosed ±0.002 pH unit repeatability and more importantly, the CO_2_ interference has been suppressed. We would comment that this work provides the ultimate approach to improving PEDOT-based SC materials.

Compared with relatively hydrophilic bulk CPs of PEDOT and PPy, POT is a highly hydrophobic CP. Ivaska et al. introduced POT [28] as the SC for Ca^2+^ sensing in 1994. The results showed a standard potential (259.3 ± 1.3 mV) and small drift (0.23 mV/day), which were comparable with conventional Ca-ISEs. Since POT is highly hydrophobic, the water-layer effect should be significantly suppressed [29]. However, the reported analytical performances are contradictions. For example, Lindner’s group prepared POT-based K^+^-SC-ISEs, displaying 1.4 mV/h drift [30]. After doping with a 7,7,8,8-tetracyanoquinodimethane (TCNQ/TCNQ^−^) redox couple, the drift decreased to 0.1 mV/h. We speculate that it might attribute to the high resistance of POT, leading to a quasi-reversible (POT/POT^n+^) particularly for the thicker POT. In addition, the light-sensitivity for POT compared with other CPs is the most challenging. It should be noted that Bakker’s group has established voltammetry-based potentiometric ion sensing by using the ET of POT coupling with the IT of target ions [31,32,33,34,35,36,37], and first proposed POT-based transducer for both anion and cation sensing by electrochemical switching [38]. PANI has also been a widely used CP for SC-ISEs by virtue of its easy electrochemical synthesis and excellent electronic/ionic conductivities. A drawback is the pH-sensitivity. Liu et al. reported a composite PANI/PMMA (poly(methyl methacrylate)) to reduce this pH-effect [39]. Numerous efforts for functionalization of PANI composites [40,41,42,43,44] have been devoted to further enhancing the analytical performance. Other CPs, for example, polyazulene [45,46], ferrocene-functionalized poly(vinyl chloride) (PVC) [47] are also proposed for the SC-ISEs.

**Figure 4:**
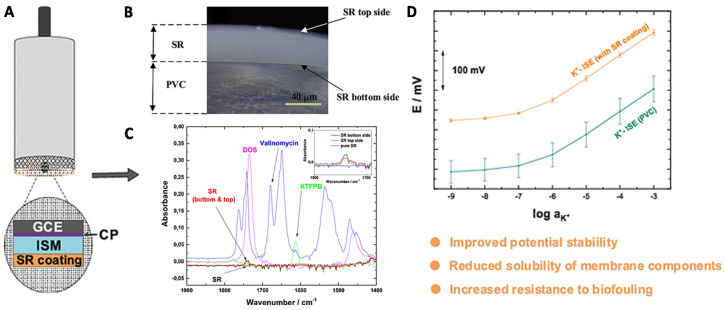
Suppress of the leaking of ISM components. (**A**) A CP-based SC-ISE by coating the silicon rubber (SR) on the ISM layer. (**B**) A photograph of the SR on the poly(vinyl chloride) (PVC)-based ISM. (**C**) Fourier transform infrared (FTIR) spectra analysis of the SR layer (bottom and top) in comparison with standard ISM components. The ISM components were observed in both bottom and top SR (typical dioctyl sebacate, DOS). (**D**) The K^+^-response of SR-coated SC-ISEs compared with an uncoated one. Reprinted with permission from [49], Copyright (2019) American Chemical Society.

In addition to the SC materials, the leaking of ISM components is another daunting challenge for the SC-ISEs. The ISM is in fact a plasticized organic solid membrane. The membrane components face the risk of leaking into the solution. Michalska et al. experimentally observed the spontaneous partition of POT into the ISM by ultraviolet/visible (UV/vis) and fluorescence microscopy [48]. The POT was distributed into the whole ISM and the partition ratio reached 0.5% w/w. Lisak and coworkers recently reported an approach by coating an outer layer of inert silicone rubber (SR) on the ISM layer to reduce the leaking [49] (Figure 4). Their experimental results demonstrated that SR coating did not affect the selectivity and improved the analytical performances remarkably. The potential stability of standard potential increased from ±35.3 mV to 3.5 mV (Figure 4D). The Fourier transform infrared (FTIR) spectra demonstrated that partial ISM components gradually diffused into SR layer (Figure 4C). Since the lower solubility of ISM components in the SR, the leaking is reduced, leading to an enhanced stability. Moreover, the SR increased the resistance to biofouling which would be beneficial to the practical measurement in a complicated environment, for example in sea water.

### 3.2. Carbon Materials

Compared with redox CPs, carbon materials emerged for SC materials by virtue of their high conductivity and chemical inertness to environment (gas and light) and redox species. Carbon-based SC materials generally rely on the EDL capacitance response mechanism. In principle, the GC directly coated with ISM like CWEs can be also recognized as the EDL-capacitance ISEs since the GC electrode acts as both solid contact and substrate. An EDL formed between SC and ISM layers. However, the GC electrode shows very small EDL capacitance resulting in the large potential drift according to Equation (6). Therefore, increasing the EDL capacitance is the core subject for the carbon-based SC-ISEs.

**Figure 5:**
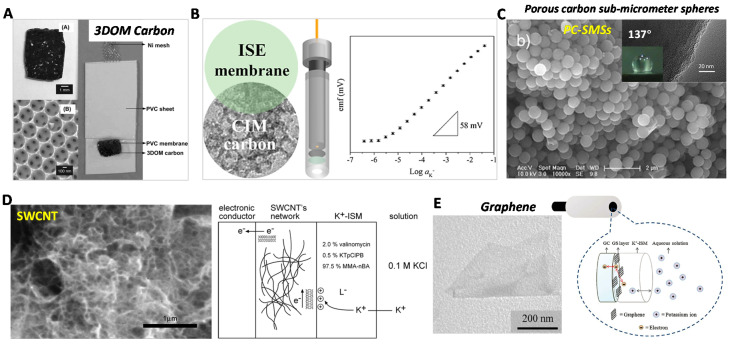
Carbon-based SC-ISEs. (**A**) High-surface 3D-ordered microporous (3DOM) carbon as a SC transducer for SC-ISEs. Reprinted with permission from [50], Copyright (2007) American Chemical Society. (**B**) Colloid-imprinted mesoporous (CIM) carbon with higher surface area as a SC transducer for SC-ISEs. Reprinted with permission from [52], Copyright (2014) American Chemical Society. (**C**) The porous carbon sub-micrometer spheres (PC-SMSs) for superhydrophobic SC transducer with the contact angle up to 137°. Reprinted with permission from [53], Copyright (2015) Elsevier. (**D**) Single-wall carbon nanotube (SWCNT)-based SC-ISEs and the illustrated EDL capacitance response mechanism. Reprinted with permission from [54], Copyright (2008) American Chemical Society. (**E**) The chemically prepared reduced graphene oxide (RGO) as SC transducer for SC-ISEs. Reprinted with permission from [55], Copyright (2012) Royal Society of Chemistry.

In 2007, a 3D-ordered microporous (3DOM) carbon was reported by Stein and Bühlmann groups as the SC material for SC-ISEs [50] (Figure 5A). Owing to the high surface area, the 3DOM carbon-based SC-ISEs showed a low potential drift of 11.7 μV/h. However, the controlled experiments by using highly oriented pyrolytic graphite (HOPG) as the SC disclosed 77 μV/h drift, which demonstrated the high EDL capacitance stabilized mechanism. As expected, there were no gas and light interferences owing to the chemical inertness of carbon materials. After the first introduction of 3DOM carbon as the SC material, they further discussed the effect of architecture and surface chemistry of 3DOM carbon on the ISE performances [51]. The untemplated carbon-based SC-ISEs showed large potential drift further indicating the significance of high EDL capacitance. Meanwhile, the surface oxidized 3DOM carbon with oxygen-containing groups exhibited higher EDL capacitance but the water-layer exists due to the hydrophilic groups. The potential drift was ca. 29 μV/h higher than the unoxidized 3DOM carbon. This work indicates the balance between capacitance and hydrophobicity.

In addition to macroporous carbon, Stein and Bühlmann et al., in 2014 introduced the colloid-imprinted mesoporous (CIM) carbon [52] as the SC material (Figure 5B). The CIM carbon showed nearly 10 times higher capacitance than the 3DOM carbon. The potential drift was further reduced to 1.3 μV/h and the standard derivation of E^0^ was only 0.7 mV. Niu’s group in 2015 developed porous carbon sub-micrometer spheres (PC-SMSs) [53] for SC-ISEs (Figure 5C). The PC-SMSs featured a high hydrophobicity (contact angle: 137°) and high capacitance (12 mF). The PC-SMSs based K^+^-SC-ISEs exhibited 3DOM carbon comparable intermediate-term stability (14.9 μV/h) and excellent long-term stability (ca. 7 mV drift over two months).

Another two representative carbon nanomaterials including carbon nanotubes and graphene have been also explored as the SC materials. In 2008, Rius first introduced single-wall carbon nanotube (SWCNT) [54] to fabricate SC-ISEs (Figure 5D). Nernstian slope and short response time were achieved. Owing to the hydrophobicity of SWCNT, the water-layer was not observed. More importantly, they illustrated in detail that the mechanism can be explained as EDL capacitive coupling [56]. The charged ions are accumulated on the ISM side of the SWCNT/ISM interface, while electrons are located on the SWCNT side, leading to charging/discharging capacitive process. In addition to the cation’s sensors, multi-wall carbon nanotube (MWCNT)-based SC-ISEs were reported for anion detection in real environmental samples by the Bakker and Crespo groups [57]. The medium-term stability and interference test were much better than the CPs-based SC-ISEs (like POT).

Niu’s group in 2011 introduced graphene sheets [55] as SC materials for K^+^-SC-ISEs (Figure 5E). The graphene films were chemically prepared by the reduction of graphene oxide (RGO). Measurements showed an improved water-layer test result and potential stability compared with CWEs. However, the performances particularly for the stability were lower than CNTs and other carbon materials. We attributed this to the existence of hydrophilic surface functional groups (R-COOH, R-OH) on the RGO, leading to a risk of the formation of water layer like the oxidized 3DOM carbon. Wu et al. reported the RGO-based SC-ISEs almost at the same time, which showed the Nernstian response and a low detection of 10^−6.2^ M K^+^ sensing [58]. Liu et al. modified the graphene oxide by 2-aminowthanethiol and then reduced it to 2-aminoethanethiol functionalized reduced graphene oxide (TRGO) [59]. TRGO could be self-assembled on gold substrate through cohesive Au–S interaction. The cationic and anionic sensing have verified that TRGO-based SC-ISEs have better long-term stability performance under continuous flowing system over two weeks. For the response mechanism, Riu and coworkers demonstrated an EDL capacitive model like other CNTs for graphene-based SC-ISEs [60]. 

### 3.3. Other Nanomaterials

**Figure 6:**
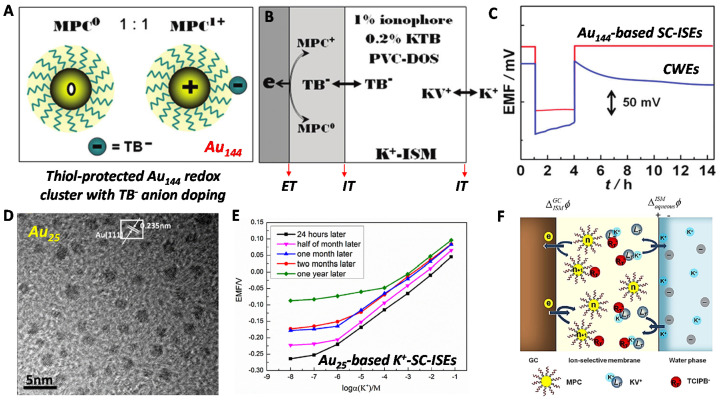
Au nanoclusters-based SC-ISEs. (**A**–**C**) Au_144_ redox nanocluster based-SC-ISEs. (**A**) Reduced and oxidized states of thiol monolayer-protected Au clusters (MPCs) (Au_144_) doped with tetrakis(4-chlorophenyl) borate (TB^−^) anion (MPC^0^/MPC^+^TB^−^). (**B**) Au MPCs-based SC-ISEs with well-defined phase interfacial potential definition. (**C**) Water-layer test of Au_144_-based SC-ISEs. Reprinted with permission from [61], Copyright (2012) American Chemical Society. (**D**,**E**) Au_25_ redox nanocluster based-SC-ISEs. (**D**) Transmission electronic image (TEM) of the Au_25_ synthesized by optimized one-phase reduction procedure. (**E**) The Au_25_-based SC-ISEs for K^+^-response to examine its long-term stability. Reprinted with permission from [62], Copyright (2016) Elsevier. (**F**) Au MPCs-based single-piece SC-ISEs through mixing the SC and ISM phase. Reprinted with permission from [63], Copyright (2016) Elsevier.

In addition to the CPs and carbon materials, a few promising SC materials have been proposed. Niu’s group in 2012 developed thiol monolayer-protected Au clusters (MPCs) [61] with tetrakis(4-chlorophenyl) borate (TB^−^) anion doping for the SC (Figure 6A–C). It should be noted that the Au MPCs is a macromolecule with specific molecular weight. More importantly, the Au MPCs have redox activity and the oxidization and reduced states (MPC^+1/0^) to form the redox buffer. The GC/SC interface is controlled by the ET of MPCs with well-defined phase interfacial potential (Equation (2), Figure 6B). The doped hydrophobic TB^−^ anion existed in both ISM and SC, performing a reversible IT at the SC/ISM interface. The SC/ISM interfacial potential can be described by Equation (3). The ISM/aq interfacial potential is clear (Equation (4)). Thus, the Au MPCs-based SC-ISEs has clear and well-defined potential definition. The water-layer test demonstrated no water film existed owing to the superhydrophobic thiol protection. The stability showed a deviation of 10.1 ± 0.3 μV/h over continuous monitoring of 72 h (Figure 6C). The standard derivation of E^0^ reproductivity was evaluated about ±0.8 mV. This work in one hand proposed redox and superhydrophobic Au MPCs as the SC for SC-ISEs. More importantly, the crucial point is the MPC^+1/0^ redox buffer and TB^−^ anion doping that offer well-defined phase interfacial potentials.

Regarding to the complex synthesis and low-yield of Au MPCs, Niu’s group further proposed one-phase reduction procedure to prepare Au_25_ cluster with high yield [62] (Figure 6D). The Au_25_-based SC-ISEs showed a long-term stability over half a month (Figure 6E). Furthermore, they fabricated a single-piece SC-ISEs by Au MPCs solid contact to simplify the electrode structure [63] (Figure 6F). A Nernstian response resulted, and the selectivity was not affected. The long-term stability can also last over two weeks.

**Figure 7:**
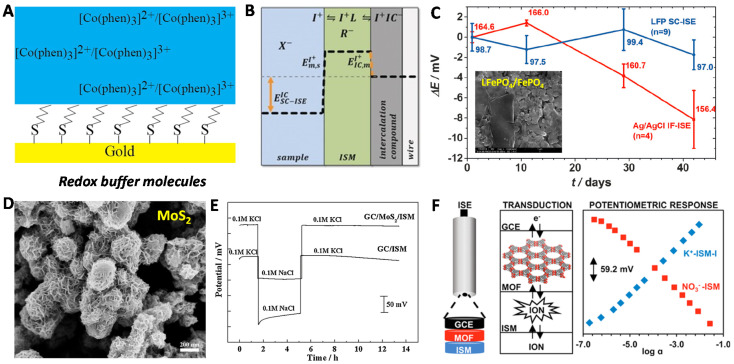
A few other representative SC materials. (**A**) Co(II)/Co(III) complex redox buffer-based SC-ISEs. Reprinted with permission from [64], Copyright (2013) American Chemical Society. (**B**,**C**) Lithium-battery materials of LiFePO_4/_FePO_4_-based SC-ISEs. Reprinted with permission from [70], Copyright (2016) John Wiley and Sons publications. (**D**,**E**) MoS_2_ nanomaterials was used for the EDL-type solid contact. SEM image of MoS_2_ (**D**) and water layer test (**E**). Reprinted with permission from [71], Copyright (2016) Elsevier. (**F**) Metal–organic frameworks (MOFs)-based SC-ISEs. Reprinted with permission from [73], Copyright (2018) American Chemical Society.

Like the Au MPCs^+1/0^ redox buffer, Bühlmann et al., in 2013 reported redox Co(II)/Co(III) complex molecules on the thiol-modified Au electrode for the SC-ISEs [64] (Figure 7A). The Co coordination compounds were also doped with TPFPhB^−^ counter ion to form a well-defined phase boundary potential at the SC/ISM interface determined by TPFPhB^−^ transfer. The response mechanism is the same with the MPCs-based SC-ISEs. It showed a small standard derivation of E^0^ of 1.0 mV over two weeks. Other Co complexes were reported for the SC, which further demonstrated the redox-buffer stabilized concept [65,66,67,68]. In addition to Co complexes, tetrathiafulvalene (TTF) and its radical salts have also constituted the redox buffers for SC-ISEs [69]. Schuhmann and coworkers presented an intercalative compound (LiFePO_4_/FePO_4_) [70] redox buffer for ion-to-electron transduction (Figure 7B,C). When ion flux comes through the membrane, there is a reversible ion exchange (Fe^Ⅱ/^^Ⅲ^) accompanied by Li^+^ intercalation/deintercalation. This mechanism provided a well-defined redox interfacial potential and large redox capacitance (Figure 7B). In this configuration, a potential drift of −1.1 ± 1.4 μV/h over 42 days was reported, as well as a standard E^0^ deviation of ±2.0 mV (Figure 7C). This example discovered the growing family of classical lithium battery materials for SC-ISEs.

For the EDL capacitance-type SC-ISE, Qin et al. presented a 3D molybdenum sulfide (MoS_2_) [71] nanoflower-based SC-ISEs sensor for K^+^ detections (Figure 7D). The SC material was produced by hydrothermal method, and directly drop-casted on GC electrode. The MoS_2_-based SC-ISEs showed good Nernstian response and no water layer was observed (Figure 7E). This group further reported an oxygen vacancy MoO_2_ for SC-ISEs [72]. The oxygen vacancy increased the electronic conductivity and the EDL capacitance, leading to high stability.

In addition to the high surface area carbon materials, metal–organic frameworks (MOFs) [73] with high porous characteristic were first implemented as ion-to-electron transducer by Mirica and coworkers (Figure 7F). The material was synthesized by interconnection of 2,3,6,7,10,11-hexahydroxytriphenylene-HHTP and Ni, Cu, or Co nodes in a Kagome lattice. The prepared electrode exhibited large bulk capacitance (204 ± 2 μF) and good potential stability (drift of 11.1 ± 0.5 μA/h).

The above MPCs, Co complex and LiFePO_4_ in principle represent the redox capacitance response mechanism. The MoS_2_, MoO_2_ and MOFs can be assigned to the EDL capacitance-based SC-ISEs. Exploring SC transducer materials is still an attractive topic in the field of potentiometric sensing. The realization of SC-ISEs with standard E^0^ reproducibility and long-term stability remain urgent requirements.

## 4. Wearable Sensors

The wearable sensor has become a new concept of ion-selective devices [1,4,6,7,8,9,74]. As proposed, biomedical diagnostics are experiencing a change from labor-based implements to portable devices. Wearable ion sensors are one of the portable technologies for healthcare, sports performance monitoring and clinical diagnosis. Compared with traditional LC-ISEs, SC-ISEs reveals the advantages for wearable sensors that can be miniaturized and integrated for continuously detecting the ions in body fluids like sweat, interfacial fluid or saliva in a non-invasive way. Many efforts have contributed to combining SC-ISEs with wearable electronics for real-time bio-fluids monitoring by using garment-, patch-, tattoo-, or sweatband-based objects. In this section, we will introduce recent achievements in the fabrication of SC-ISEs for wearable ion sensors.

### 4.1. Sweat Ion Detection

Sweat contains abundant chemical information that could evaluate the human body’s health state at a biomolecular level according to the medical research. Among which, Na^+^ and Cl^-^ are the main ion species ingredients. Beyond that, metabolites, acids, hormones, small proteins and peptides are also the major components of sweat. Their wide fluctuations in an extensive range indicate certain changes in human-body condition. For example, excessive loss of sodium and potassium ions in sweat could result in hyponatremia, hypokalemia, muscle cramps or dehydration. Lithium is an important mood stabilizing drug. They are important biomarkers to evaluate electrolyte balance. Traditional methods for sweat collecting and analyzing separately, would lead to inaccurate analysis due to sample contamination or evaporation in collection process. Real-time collection and detection appear a promising way to achieve accurate measurements.

**Figure 8:**
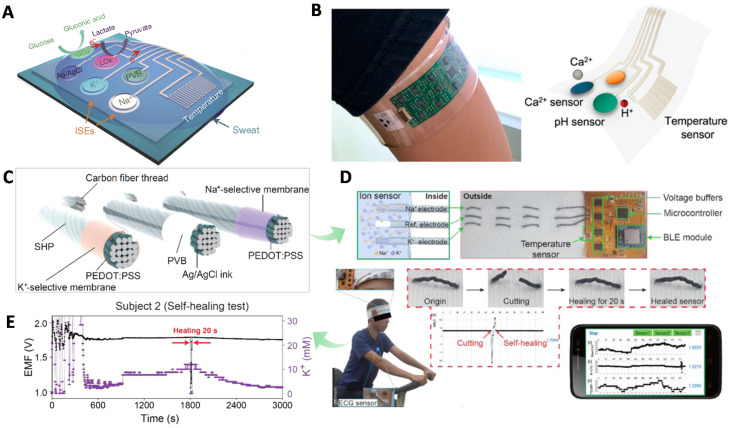
CPs-based wearable SC-ISEs for skin sweat ion sensing. (**A**) An integrated multi-parameter electrochemical wearable sensor involving Na^+^ and K^+^-SC-ISEs, glucose, lactate and temperature. The PEDOT was used for the SC transducer. Reprinted with permission from [75], Copyright (2016) Springer Nature. (**B**) PEDOT-based wearable SC-ISEs for pH and Ca^2+^ sensors. Reprinted with permission from [76], Copyright (2016) American Chemical Society. (**C**–**E**) PEDOT-based self-healable SC-ISEs for wearable senor. Reprinted with permission from [77], Copyright (2019) American Chemical Society.

***CPs-based wearable SC-ISEs.*** Gao and co-workers reported a mechanically flexible fully integrated sensor for simultaneous sensing multiple biomarkers in sweat [75] (Figure 8A). Na^+^ and K^+^ sensors were the SC-ISEs with PEDOT(PSS) as the SC, which was synthesized by galvanostatic electrochemical polymerization. The assembled sensor array was fabricated on a flexible polyethylene terephthalate (PET) substrate combined with commercially available integrated-circuit technologies, which allowed for the data visualization in the meantime. This integration technique has established the connection between signal generation, transduction, and visualization without any external multimeter. The chronoamperometric response curves of each component (Na^+^, K^+^, glucose, lactate, temperature) were performed separately in different analyte solutions, in which the results showed good potential stability. On-body physical monitoring was also performed. All the indicators exhibited normal characteristics over time, consistent with ex situ measurement results. Besides, the flexible PET substrate meets the requirements of skin friendly. This work opens a breakthrough for the wearable and integrated multiparameter sensors end exhibits the promising real-time on-body monitoring. After that, Javey et al. further extended this concept for the detection of Ca^2+^ and pH in sweat [76] with PEDOT(PSS) as ion-electron transducer (Figure 8B). Ca^2+^ is an important ion related to human metabolism and mineral balance. Ca^2+^ deficiency to a large degree may lead to diseases such as myeloma. They found an interesting result that the Ca^2+^ concentration increases with the decrease of pH in sweat.

Yoon et al., very recently proposed a self-healable wearable ion sensor for real-time sweat monitoring [77] (Figure 8C–E). Typically, carbon fiber was used as substrate and PEDOT(PSS) was used as the SC transducer. K^+^ and Na^+^ membrane cocktails were dip-coated on the surface of them. On the top of ISM was coated by poly(1,4-cyclohexanedimethanol succinate-*co*-citrate) (PCSC), a supramolecular self-healing polymer (SHP, Figure 8C). The mechanical flexibility of carbon fiber enables the sensor to be attached on many kinds of textile substrates, such as body tattoo, headband, wristband, etc. As shown in Figure 8D, the healed sensor was worn in a tester’s head and testing results were real-time checked on a handphone through the integration of a wireless circuit board. The on-body monitoring showed a real-time and accurate reflecting of the user’s conditions. When user started cycling, his heart beat fast accompanied with the increase of skin temperature, and then the ion-sensing signal of Na^+^ began to be observed and increased rapidly and kept steady, while the signal of K^+^ underwent a gradual increase during cycling. The self-healing ability of the sensor was examined during breaks of the on-body sweat test. After 20 s waiting, the sweat sensor went back to work normally, as before (Figure 8E). This work opens the possibility of a wearable sensor unconstrained by mechanical failure.

**Figure 9:**
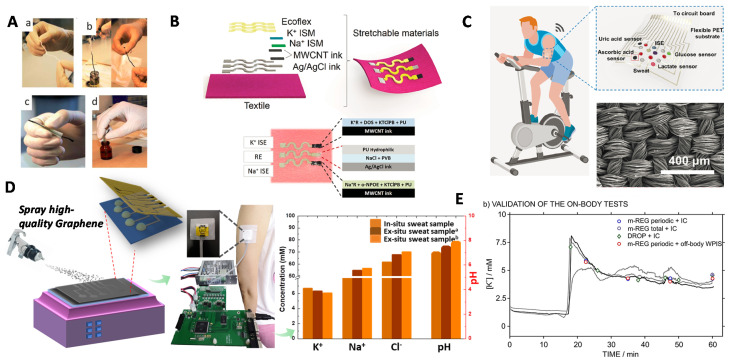
Carbon-based wearable SC-ISEs for skin sweat ion sensing. (**A**) SWCNT-based flexible SC-ISEs by using cotton yard as flexible substrate. Reprinted with permission from [78], Copyright (2013) Royal Society of Chemistry. A series of preparation including SWCNT ink and ISM dipping as shown in (**a**–**d**). (**B**) Multi-wall carbon nanotube (MWCNT) textile-based SC-ISEs for Na^+^ and K^+^ sensing. Reprinted with permission from [80], Copyright (2016) John Wiley and Sons publications. (**C**) Carbon textile-based sensor array for multiparameter analysis. Reprinted with permission from Science Advances [81], Copyright (2019) American Association for the Advancement of Science. (**D**) High-quality graphene-based wearable SC-ISEs for multichannel ion sensing including K^+^, Na^+^, Cl^−^ and pH [82]. (**E**) The suggested protocol for on-body measurement. It should be noted that the importance of calibration of SC-ISEs to assure the accuracy of real-time analysis. Reprinted with permission from [83], Copyright (2019) American Chemical Society.

***Carbon-based wearable SC-ISEs*.** As discussed in Section 3, in addition to CPs, carbon-based materials are another type of SC-ISEs. As early as 2013, Andrade and coworkers reported a prototype of SWCNT-based wearable SC-ISEs [78] since SWCNT has been demonstrated as an effective EDL-capacitance ion-to-electron transducer [54] in 2008. The authors used the cotton yarns as the flexible substrate filled with SWCNT ink and then coated with ISM (Figure 9A). The sensors showed similar performances to the lab-made SC-ISEs (like the GC substrate). They integrated the sensor on a band-aid and worn on a human model for a proof-of-concept wearable sensing by manually injecting K^+^ solution. Wang et al. fabricated epidermal tattoo-based wearable SC-ISEs for non-invasive sweat Na^+^ monitoring by using carbon ink as the SC layer [79]. The sensor can undergo bending and stretching. The sensor was integrated with a wireless signal transduction which was useful for the on-body measurements. In addition to flexible substrates like PET, paper and tattoo, they further reported a textile-based stretchable SC-ISE for both Na^+^ and K^+^ sensing by using MWCNT as the SC [80] (Figure 9B). It should be noted that the polyurethane was used instead of PVC for the ISM to improve the mechanical stress and biocompatibility. The sensors could bear 100% strain, 180° bending, crumpling and washing. Zhang et al. reported a carbon textile-based sensor array for real-time sweat monitoring including both ions and another four types of biomarker [81] (glucose, lactate, ascorbic acid and uric acid) (Figure 9C). The N-doped carbon solid-contact was derived from silk fabric. The sensor was integrated with signal collection and transmission via a mobile phone, which realized real-time on-body analysis.

Niu’s group recently reported high-quality graphene-based multichannel wearable SC-ISEs [82] for sweat ion monitoring including Na^+^, K^+^, Cl^−^, and pH (Figure 9D). Compared with reduced graphene oxide (RGO), the high-quality graphene was synthesized by intercalation of graphite. The conductivity and hydrophobicity were much higher than RGO. The flexible paper substrate was superhydrophobic coated by CF_3_(CF_2_)_7_CH_2_SiCl_3_ (C_10_^F^), leading to a water contact angle of 147°. The superhydrophobic substrate effectively prohibited water-layer formation. The flexible sensor showed great structural integrity after being bent for 300 cycles. The real-time on-body monitoring results showed 90% accuracy compared with results verified by ex-situ analysis. Crespo et al. fabricated a four-channel wearable SC-ISEs by using MWCNT as the ion-to-electron transducer [83] (Figure 9E). In this work, importantly, the authors proposed a protocol for the on-body sweat analysis to assure the data validation. They suggested a double validation strategy, i.e., twice off-body measurements before and during on-body analysis. Before on-body measurements, the sweat was measured by wearable SC-ISEs for ex situ analysis. During the on-body measurements, the sweat was sampled at intervals (e.g., every 12.5 min) for comparing analysis by other equipment, for example, ionic chromatography (IC), inductively coupled plasma mass spectrometry (ICP-MS) and pH meter. Moreover, the SC-ISEs should be also calibrated twice before and after on-body measurements. This protocol is highly recommended, which is usefully for evaluating the performance of the wearable SC-ISEs.

**Figure 10:**
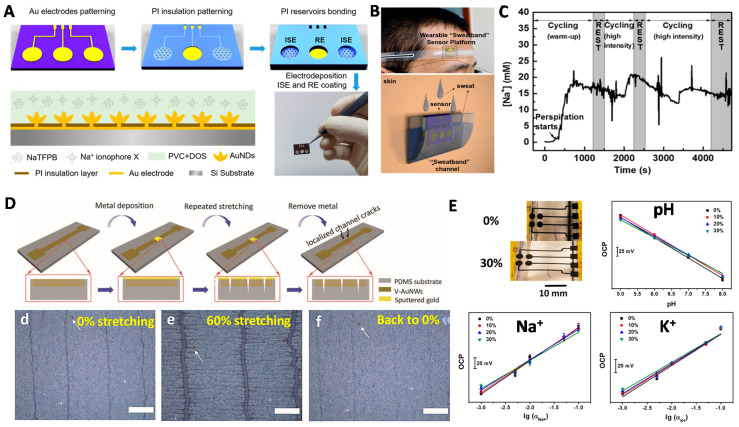
Au nanomaterials-based wearable SC-ISEs for skin sweat ion sensing. (**A**–**C**) AuND array-based SC-ISEs for sweat Na^+^ sensing. (**A**) The preparation procedure for AuND-based SC-ISEs by photolithography technique. (**B**) A schematic for the SC-ISEs on-body measurement. (**C**) Real-time analysis of sweat Na^+^ during cycling and rest states. Reprinted with permission from [84], Copyright (2017) American Chemical Society. (**D**,**E**) Gold-based vertically aligned nanowires (V-AuNWs)-based stretchable SC-ISEs for Na^+^, K^+^ and pH sensing. (**D**) The preparation of V-AuNWs on PDMS film and corresponding optical images for observing the stretching. Scale bar: 200 μm. Reprinted with permission from [85], Copyright (2019) John Wiley and Sons publications. (**E**) The Na^+^, K^+^ and pH sensing from 0% to 30% stretching. Reprinted with permission from [86], Copyright (2020) American Chemical Society.

***Au-based wearable SC-ISEs.*** With respective to CPs and carbon materials, Au materials disclose more biocompatibility. Zhang et al. recently developed a wearable SC-ISEs sensor based on gold nanodendrite (AuND) array [84] (Figure 10A–C). The AuND array was prepared by electrodeposition on a microwell array patterned chip. Different surface areas of AuND array were fabricated and tested. The results showed that AuND with larger surface area (7.23 cm^2^) exhibited enhanced potential stability. Obviously, the stable performance was improved by increasing the surface area of the SC, leading to higher EDL capacitance. The sensors performed an on-body measurement (Figure 10B). The results showed that a steady Na^+^ response was observed after ca. 15 min warm-up (Figure 10C). During the cycling/rest test, the Na^+^ level responded periodically.

For wearable sensors, the device may often endure with strains. Cheng et al. recently fabricated a stretchable sensor array with gold-based vertically aligned nanowires (V-AuNWs) as the solid contact material for multimodal sweat monitoring including Na^+^, K^+^ and pH (Figure 10D,E) [85,86]. The SC-ISEs array was prepared by using PDMS as flexible substrate. The assembled SC-ISEs tattoo was integrated with a flexible printed circuit board (PCB) to achieve wireless real-time monitoring. The durability of sensors was tested by stretching from 0% to 30% (Figure 10E). The electrode maintained stable potential response.

### 4.2. Ion Detection in Other Body Fluids

Interstitial fluid is another body fluid that contains a large amount of ingredients related to the human body’s health condition. However, the sampling process involves inserting a needle into skin [87] and waiting for the results analyzed by a complex laboratory instrument. This unpleasant process may lead to some complications. It is desirable to develop a device to monitor human physical condition simply and painlessly, especially for patients who take medicine under administration, diabetes patients for example.

**Figure 11:**
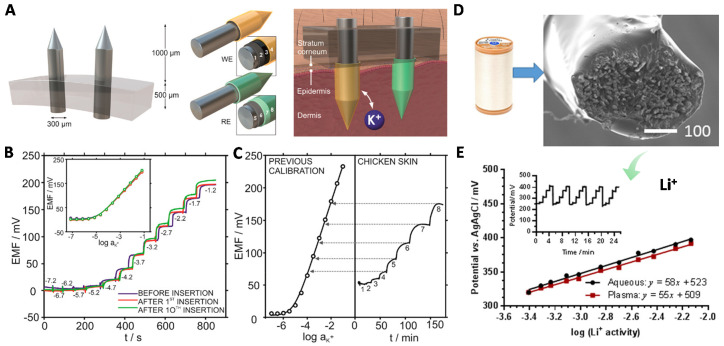
Wearable SC-ISEs for ion detection in interstitial fluid. (**A**–**C**) Microneedle-based SC-ISEs for K^+^ analysis in skin interstitial fluid. (**A**) Illustration of microneedle patch including working electrode (WE) and reference electrode (RE). For the WE, the bare microneedle was coated carbon, f-MWCNTs and K^+^-ISM. For the RE, the bare microneedle was coated Ag/AgCl; and poly(vinly butyral) membrane and polyurethane. (**B**) K^+^ response before and after insertion into the animal skin. (**C**) Ex vivo K^+^ measurement in chicken skin with calibration. Reprinted with permission from [88], Copyright (2019) American Chemical Society. (**D**,**E**) A cotton fiber-based SC-ISEs for Li^+^ sensing in the human plasma. (**D**) A SEM image for the cotton-based SC-ISEs. (**E**) Li^+^-response in aqueous solution and human plasma. The inset shows the time traces for Li^+^ response. Reprinted with permission from [89], Copyright (2018) American Chemical Society.

Recently, Crespo and co-workers presented a microneedle patch for intradermal detection of potassium in interstitial fluid [88]. The microneedle-based potassium-selective electrode was fabricated with functionalized multiwalled carbon nanotubes (f-MWCNT) and potassium membrane cocktail (Figure 11A–C). These microneedle-based electrodes were fixed in an epidermal patch which was designed to be suitable for insertion into the skin and reached the interstitial fluid in human dermis (Figure 11A). The sensor showed high repeatability in which the potentiometric calibration curves were nearly overlapped before and after 10 insertions (Figure 11B). They performed ex vivo experiments on a piece of chicken skin which was exposed to increasing changes in potassium concentration in an external solution (artificial interstitial fluid) (Figure 11C). As observed, it took ca. 30 min to reach a steady potential response. The difference of concentration between calibration and testing object was attributed to skin state, fat content, individual variation, and the storage condition for the ex vivo test. These factors reveal the challenges for interstitial ion detection.

Considering the difference of ion concentrations measurements inside and outside of the skin, Crean et al. fabricated a conductive cotton fiber-based SC-ISEs for realizing extraction and analysis simultaneously combined with reverse iontophoresis [89] (Figure 11D,E). This can also be viewed as another kind of in-situ monitoring with simplified approach. The composition of interstitial fluid and human plasma have great in common, except for a higher protein content in the latter. Considering the difficulty of commercial acquisition of interstitial fluid, human plasma was used in this work to test the Li^+^ response. The electrodes were used in plasma solutions without condition. The EMF response in plasma showed nearly overlapped results compared with aqueous solution (Figure 11E). 2–3 mV decrease compared with that in LiCl solution, because of the interference of Na^+^. This work opens alternative approach for detecting Li^+^ drug in human plasma.

Tears, urine and saliva are also the human biological fluids for non-invasive monitoring in SC-ISEs-based wearable sensors. For example, Riu et al. fabricated a disposable SC-ISEs sensor for detecting the K^+^ in human saliva [90]. The modified hydrophobic SWCNT was used as the transducer. The K^+^ concentrations of five different saliva samples showed good consistence compared with the results of atomic emission spectrometry measurements. Javey et al. tested Ca^2+^ and pH in urine and tears off-body [76]. The results showed near-Nernstian response. Andrade and co-workers reported an SC-ISEs-based sensor with enhanced recognition property for determining creatinine in diluted urine samples [91]. Overall, these biological fluids also contain abundant health biomarkers, but they might be more suitable for off-body analysis.

## 5. Conclusions and Outlook

In this review, we have briefly illustrated the development of SC-ISEs including response mechanisms, representative transducer materials and recent achievements in the fabrication of flexible and wearable sensors. There have been undeniable breakthroughs in the field of SC-ISEs aiming at constructing potentiometric wearable sensors with flexibility, mechanical stability, signal stability, and integration, involving novel SC materials and deeper understanding of their response mechanisms. Through persistent exploration of new solid transducer materials, improvements in high signal stability and good reproducibility have been obtained. The detection limit has been lowered and water uptake has been reduced through effective approaches to control the ion-to-electron transducers.

Nowadays, on-body analysis is popular since body fluids provide multiple forms of health-related information. SC-ISEs for wearable sensors monitor an individual’s health condition. Remarkable achievements have resulted in improving stability and useful life and flexibility, by incorporating excellent SC transducers with suitable substrates in the form of textiles, tattoos, headbands, etc. The new non-invasive wearable platform shows promising prospects in variety of healthcare-related areas. They provide low-cost, real-time, and precise detection. As we discussed in the final part of the review, reliable analytical method requires synergistic action. For example, flexible substrate materials are expected to adapt user’s motion movements without disturbing daily life; SC-ISEs are required to provide accurate and quick monitoring; the integrated circuit board connect with mobile platform reflect ions’ concentration or process changes over time. Considering the complex processing of human physiological biomarkers, next generation of sensing platforms are expected to explore new materials and new sensing techniques.

For the outlook of SC-ISEs, we think the following perspective is of significance:

(1) There is an urgent protocol requirement for evaluating the SC-ISEs, for example, a standard measurement protocol for the long-term stability of the potential drift. In addition, evaluation of the redox or EDL-capacitance of the SC materials should provide the specific capacitance for comparison. For the wearable sensors, a standard measure protocol should be also presented as soon as possible including calibration and data validity [83]. (2) In addition to the WE of SC-ISEs, the RE should be given more attention. Currently, polymer-based [92,93,94], ionic liquid-junction [95,96,97], and lipophilic salts modified-polymer [98,99] are the main solid-state REs. Recently, a solid-state RE of Ag/AgI based on a self-referencing pulstrode offers a new strategy [100]. (3) The leaking of ISM components is another issue that should be emphasized. SR coating provides an approach [49]. Development of new ISM membrane or making transducer with ion selectivity are plausible. (4) The biocompatibility of SC transducer and the ISM should be focused upon [101,102]. The ISM component, like the valinomycin for K^+^ ionophore, is a neurotoxin. Biocompatibility should be paid sound attention particularly in the process of practical in vivo measurements.

Over the past few decades, we have witnessed the development of ISEs from LC to SC that has miniaturized and integrated the ISEs. In addition to the wearable sensors, SC-ISEs could be further intensively explored for in vivo investigations at cell, tissue or organ level [103,104,105,106,107]. More scientific challenges and the promise of practical application provide tremendous space and the next hot-spot for the SC-ISEs.

## Appendix A

For the redox capacitance-based SC-ISEs, there are three interfaces including GC/SC, SC/ISM and ISM/aq (Figure 2A). ET occurs at GC/SC interface with well-defined phase interfacial potential (Equation (2)). For the counter ion transfer at the SC/ISM interface, the interfacial potential (Equation (3)) is derived as follows. The IT of Y^−^ at SC and ISM are equilibrium,

(A1) $$Y^{-}\left(\mathrm{SC}\right)⇌Y^{-}\left(\mathrm{ISM}\right)$$

Thus, the Y^−^ electrochemical potential, ${\bar{\mu }}_{Y^{-}}$ in SC and ISM phases is equal, i.e.,

(A2) $${\bar{\mu }}_{Y^{-}}\left(\mathrm{SC}\right)={\bar{\mu }}_{Y^{-}}\left(\mathrm{ISM}\right)$$

According to the electrochemical potential expression,

(A3) $${\bar{\mu }}_{Y^{-}}\left(\mathrm{SC}\right)=\mu_{Y^{-}}^{\Theta \prime }\left(\mathrm{SC}\right)+RT\ln{\left[Y^{-}\right]}_{\mathrm{SC}}-F\phi \left(\mathrm{SC}\right)$$

(A4) $${\bar{\mu }}_{Y^{-}}\left(\mathrm{ISM}\right)=\mu_{Y^{-}}^{\Theta \prime }\left(\mathrm{ISM}\right)+RT\ln{\left[Y^{-}\right]}_{\mathrm{ISM}}-F\phi \left(\mathrm{ISM}\right)$$

where $\mu_{Y^{-}}^{\Theta \prime }\left(\mathrm{SC}\right)$ and $\mu_{Y^{-}}^{\Theta \prime }\left(\mathrm{ISM}\right)$ are the conditional chemical potentials in the SC and ISM phases, respectively. ϕ(SC) and ϕ(ISM) are the phase potentials in SC and ISM phases, respectively.

Combining Equations (A2)–(A4), it can be obtained the SC/ISM interfacial potential $E_{\mathrm{ISM}}^{\mathrm{SC}}$,

(A5) $$E_{\mathrm{ISM}}^{\mathrm{SC}}=\phi \left(\mathrm{SC}\right)-\phi \left(\mathrm{ISM}\right)=\frac{\mu_{Y^{-}}^{\Theta \prime }\left(\mathrm{SC}\right)-\mu_{Y^{-}}^{\Theta \prime }\left(\mathrm{ISM}\right)}{F}+\frac{RT}{F}\ln\frac{{\left[Y^{-}\right]}_{\mathrm{SC}}}{{\left[Y^{-}\right]}_{\mathrm{ISM}}}$$

When the ${\left[Y^{-}\right]}_{\mathrm{ISM}}$ and ${\left[Y^{-}\right]}_{\mathrm{SC}}$ equal the unit (like the ET of oxidation and reduction species in the Nernst equation), $E_{\mathrm{ISM}}^{\mathrm{SC}}$ represents the conditional electrode potential ΔISMSCϕO′(Y−). Thus,

(A6) EISMSC=ϕ(SC)−ϕ(ISM)=ΔISMSCϕO′(Y−)+RTFln[Y−]SC[Y−]ISM

For the K^+^ transfer at the ISM/aq interface, similar derivation like Y^−^ at SC/ISM interface can result in Equation (4) according to the K^+^ electrochemical potential equilibrium in ISM and aq phases.
